# Intradermal priming to intravesical Bacillus Calmette-Guérin in non-muscle invasive bladder cancer: A translational research and phase I clinical trial

**DOI:** 10.32604/or.2025.061812

**Published:** 2025-05-29

**Authors:** LUCIANA SABOYA, KEINI BUOSI, TIAGO SILVA, ELAINE CANDIDO, JOSIANE MORARI, LICIO A. VELLOSO, SHAHROKH F. SHARIAT, MARCUS V. SADI, LEONARDO O. REIS

**Affiliations:** 1 UroScience, School of Medical Sciences, University of Campinas, Campinas, 13083-887, Brazil; 2 Department of Urology, Federal University of São Paulo, EPM, São Paulo, 04023-062, Brazil; 3 Obesity and Comorbidities Research Center, University of Campinas, Campinas, 13083-887, Brazil; 4 Department of Urology, Comprehensive Cancer Center, Medical University of Vienna, Vienna, 1090, Austria; 5 Hourani Center for Applied Scientific Research, Al-Ahliyya Amman University, Amman, 19328, Jordan; 6 Department of Urology, University of Texas Southwestern Medical Center, Dallas, TX 75390, USA; 7 Department of Urology, Weill Cornell Medical College, New York, NY 10065, USA; 8 ImmunOncology, Pontifical Catholic University of Campinas, Campinas, São Paulo, 13060-904, Brazil; 9 UroGen, National Institute of Science, Technology and Innovation in Genitourinary Cancer (INCT), Campinas, São Paulo, 13087-571, Brazil

**Keywords:** Bladder cancer, Bacillus Calmette-Guérin (BCG), Intradermal, Immune response, Safety

## Abstract

**Objective:**

To determine the safety and the role of modulating cytokines and proteases in the immune response to intravesical Bacillus Calmette-Guérin (BCG) when primed with systemic intradermal BCG.

**Methods:**

Phase 1 and mechanistic longitudinal, prospective, single-blind randomized study (NCT04806178). Twenty-one non-muscle invasive urothelial bladder cancer patients undergoing intravesical adjuvant BCG after transurethral resection of bladder tumor (TURBT) in a teaching hospital between September 2021 and April 2023 were randomized to 0.1 mL of intradermal BCG vaccine or placebo (0.9% saline) administered 15 days before the start of intravesical BCG therapy. Blood samples were evaluated mechanistically regarding eight cytokines serum levels interferon-induced transmembrane protein 3 Gene (IFITM3), Interleukin 1 beta (IL1-BETA), interleukin-2 receptor alpha chain (IL2 RA), Interleukin 6 (IL 6), Interleukin 10 (IL 10), Tumor necrosis factor alpha (TNF-α), Interferon-β, AXL, and one protease CASPASE 8.

**Results:**

After 1 exclusion, twenty patients were randomized to intradermal BCG (n = 11) and intradermal placebo (n = 9). There was no difference in adverse effects emerging from the intravesical Onco-BCG therapy, and no difference in the expression of the cytokines and proteases analyzed between control and intervention, and over time.

**Conclusions:**

Intradermal BCG administration before intravesical application was safe, with no increase in adverse effects. It also does not seem to change the analyzed targets during the intravesical induction-phase BCG. Other immune targets should be explored in the future. The Brazilian tuberculosis-endemic status, where BCG vaccination is mandatory, might have affected the results.

## Introduction

The incidence of bladder cancer was estimated in 83,190 new cases in 2024 in the US, which makes it the 7th most diagnosed cancer among men, accounting for 0.86 female and 3.3 male deaths in 100,000 people worldwide per year, and the 9th cancer-specific cause of mortality [1,2]. For over forty years, Bacillus Calmette-Guérin (BCG) has been pivotal in treating and managing non-muscular invasive bladder cancer (NMIBC), accounting for 75% of cases [2].

Evidence shows reduced tumor progression and recurrence when intravesical immunotherapy BCG is performed. This vaccine is superior to mitomycin and other chemotherapeutics in reducing the rate of tumor recurrence by up to 32%. In addition, adding the BCG maintenance phase guarantees improved recurrence rates and can reduce the odds of progression by 27% [3,4].

However, until today, limited light has been shed on the exact mechanisms by which the bacillus, tumor cells, and the immune system interact. Evidence indicates that the innate and adaptive immune systems act on the tumor via direct injury to tumor cells by activating natural killer cells. In contrast, dendritic cells, neutrophils, macrophages, and TCD4, TCD8, and Tγδ cells may play a key role in the process [5].

Despite this condition’s high prevalence and mortality rate, the world has suffered from intermittent shortages of BCG production, be it due to the complexity and morosity of the bacillus production, the absence of technological investments in streamlining production, or even disinterest from the pharmaceutical industry, as evidenced by the low cost of the medication [6,7].

In this scenario, the only alternatives are gemcitabine and mitomycin. Despite new emerging research studies with chemotherapy drugs, tyrosine-kinase inhibitors, and immunomodulators, no drug has proven superior to intravesical BCG in reducing recurrence and disease progression rates [3].

This study aims to assess the safety of systemic priming prior to intravesical BCG administration and to analyze a strategic panel of cytokines and protease that play a key role in the immune response to microorganisms, neoplasms, and the inflammatory process, which, to our knowledge, have not yet been studied in the BCG/NMIBC context.

## Materials and Methods

A Phase 1 and mechanistic longitudinal, prospective, randomized, single-blind study (NCT04806178) was conducted at an academic institution, the Federal University of São Paulo, Brazil between September 2021 and April 2023.

After Escola Paulista de Medicina (EPM) ethics committee approval and consent (number 196/21), twenty-one NMIBC patients who had undergone transurethral resection of bladder tumor (TURBT) and were eligible for adjuvant intra-vesical immunotherapy with BCG as per National Comprehensive Cancer Network (NCCN) criteria [4] (intermediate and high-risk tumors) were randomized to 0.1 mL of intradermal BCG vaccine or placebo (0.9% saline) in the inferior insertion of the deltoid muscle on the left arm (time T0), administered 15 days before the start of intravesical BCG therapy.

The study’s inclusion criteria were subjects over 18 years old at the time of the study, anatomopathological diagnosis of non-muscle invasive pure urothelial bladder cancer, having undergone full resection, eligibility for adjuvant therapy, and signed informed consent. Childhood BCG vaccination was used as an inclusion criterion when purified protein derivative (PPD) test or Interferon Gamma Release Assay (IGRA) test results were unavailable to ensure consistency. This decision was made because Brazil is a tuberculosis-endemic country where BCG vaccination is mandatory.

Exclusion criteria were a muscle-invasive tumor, previous therapy with intravesical BCG, and ineligibility for vesical catheterization. Randomization into intradermal (ID) BCG vaccine and placebo included sex, age, and disease stage as stratification factors and was done using the website https://www.sealedenvelope.com/simple-randomiser/v1/lists (accessed on 31 March 2025).

The REDCap platform was cosen to collect and record clinical data, such as comorbidities, medication use, previous surgeries, and urinary symptoms, which were scored based on the I-PSS (International Prostate Symptoms Score). The I-PSS score was also used for women because no questionnaire was validated for both sexes [8].

**Figure 1:**
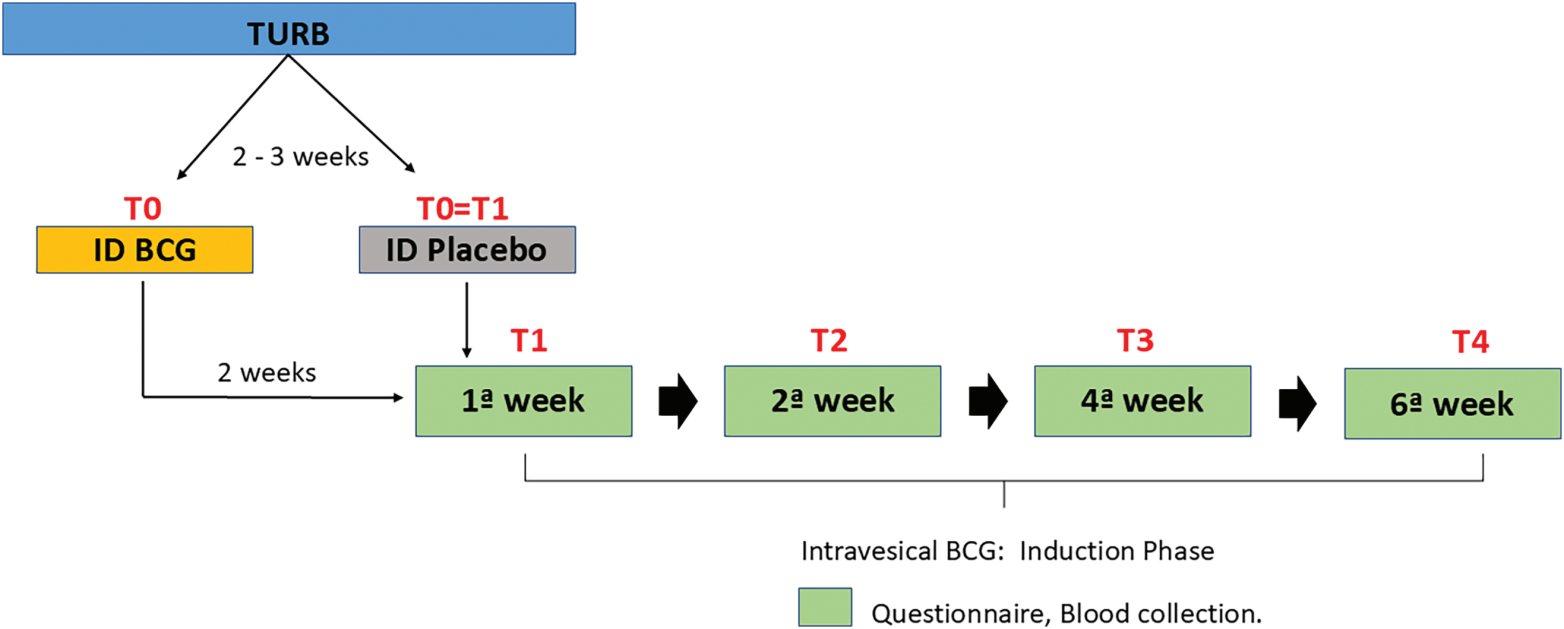
Study design.

Following randomization, patients were subject to anamnesis and blood sample collection upon admission (T0), and with every follow-up visit for the standard of care intravesical BCG 80 mg diluted in 50 mL of 0.9% saline solution maintained in the bladder for 2 h in the first (T1), second (T2), fourth (T3), and sixth (T4) weeks (Fig. 1).

To determine the safety and adverse effects, eight contexts were used: four time points and two interventions (intradermal BCG and placebo). Systemic and urinary symptoms related to intradermal and intravesical standard BCG treatment were measured at T0, T1, T2, T3, and T4 (Fig. 1). Our center also provides standardized follow-up of these patients, as well as guidance on symptoms and access to assistance in case of suspected complications.

To determine mechanistic insights, four contexts were used: two time points and two interventions (intradermal BCG and placebo). Blood samples were compared for A—time zero (no intervention, **Control**), B—fifteen days after intradermal BCG (**Intradermal BCG**), C—28–35 days after intravesical BCG in those after intradermal placebo (**Intravesical BCG**), and D—28–35 days after intravesical BCG in those primed by intradermal BCG (**Intradermal + Intravesical BCG**).

The mechanistic evaluation covered one protease, CASPASE 8 [9,10], and eight cytokines serum levels: IFITM3 [11,12], IL1-BETA [13,14], IL2 RA [15,16], IL 6 [17,18], IL 10 [19,20], TNF-α [21,22], Interferon-BETA [23,24], and AXL [25], in Table A1.

RNA was extracted from the samples using 1 mL of Trizol reagent (Cat No. 15596026, Applied Biosystems, Foster City, CA, USA), and the High-Capacity cDNA Reverse Transcription kit (Cat No. 4374967, Applied Biosystems, Foster City, CA, USA) was used for cDNA synthesis, as per manufacturer’s instructions, respectively. Then, 10 ng/μL of the cDNA, 3 μL of the LuminoCt qPCR ReadyMix (Cat No. L6669, Sigma-Aldrich, St. Louis, MO, USA), and 0.25 μL of each primer were used to quantify gene expression.

Peptidylprolyl isomerase A (Ppia), a housekeeping gene, was used as a reference for endogenous control. It is critical in developing many human cancers by regulating cell growth [26]. The primer assays are described in Table A2. The qPCR was performed in the StepOne Real-Time PCR system (Applied Biosystems, Foster City, CA, USA, EUA), and all assays were duplicated. The analysis was made using the comparative 2^-ddct method.

### Statistical analysis

A descriptive analysis of variables included absolute (n), relative (%) frequency distributions (n), average, standard deviation, median, minimum, and maximum values.

Relationships between qualitative and quantitative variables were established using the chi-square test or Fisher’s exact test, as appropriate. When data normality was present, the *t*-test was used to compare the distribution of quantitative variables in relation to clinically relevant variables. When this assumption was violated, the non-parametric Mann-Whitney test was performed.

The non-parametric Kruskal-Wallis test compared the protease and cytokines distribution in blood samples of clinically relevant interventions and time points as independent. The level of significance used was 5%. International Business Machines Corporation, New York, NY, USA, EUA, conducted statistical analyses using IBM SPSS version 28 and R version 4.3.1 software.

## Results

**Figure 2:**
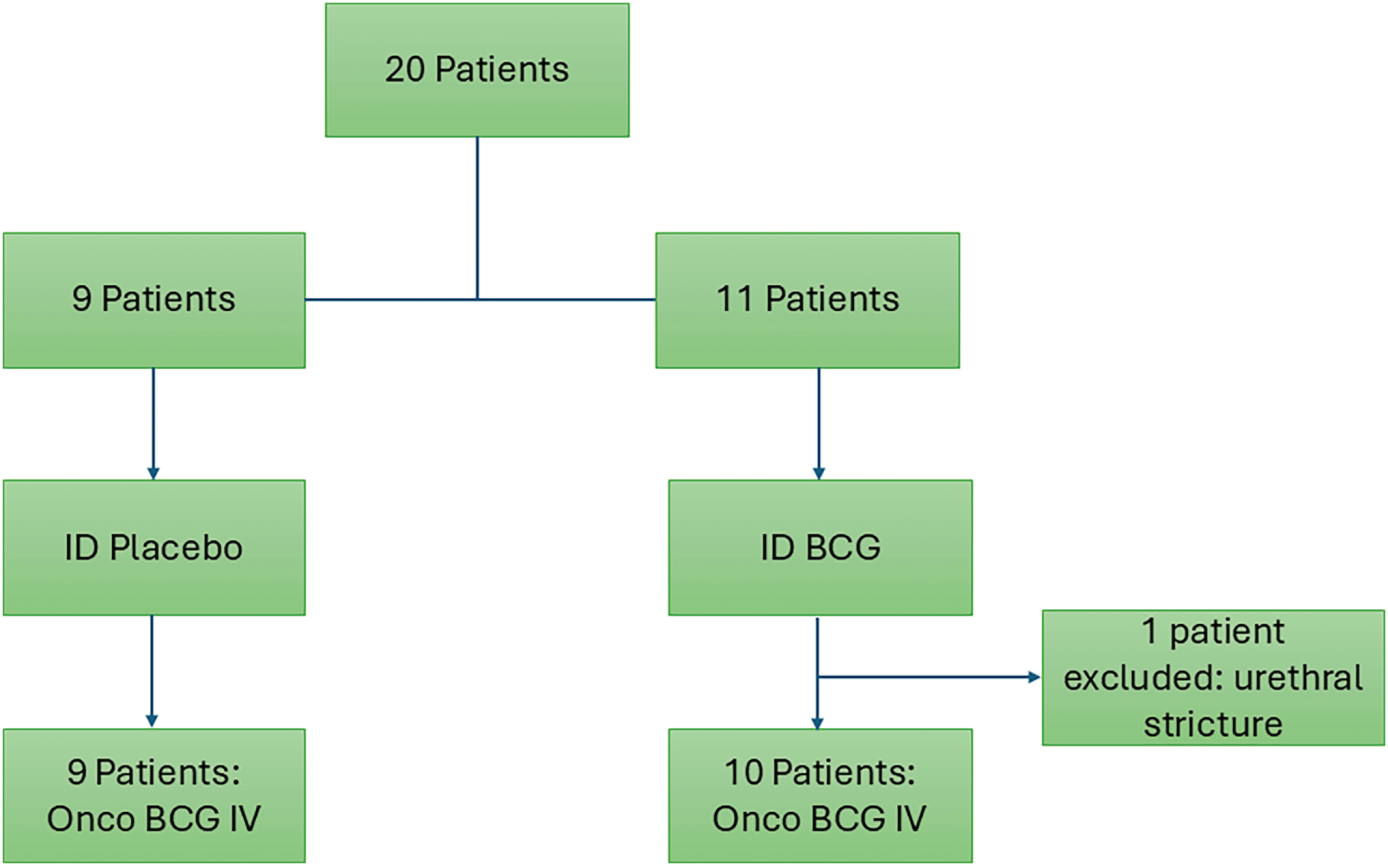
Study flow chart.

After 1 patient exclusion due to urethral stricture, twenty consecutive patients were randomized to intradermal BCG (n = 11) and intradermal placebo (n = 9), as shown in Fig. 2. Table 1 shows clinical and epidemiological characteristics.

**Table 1:** Epidemiological and clinical data of the included patients

		${Total}^{a}$	Placebo	Intradermal BCG	
		n (%)	n (%)	n (%)	*p*
*Total*		20 (100)	9 (45)	11 (55)	
*Sex*					
	*Male*	10 (50.0)	4 (44.4)	6 (54.5)	0.999
	*Female*	10 (50.0)	5 (55.6)	5 (45.5)	
*Previous tuberculosis*					
	*No*	20 (100)	9 (100)	11 (100)	
	*Yes*	0 (0)	0 (0)	0 (0)	
*Previous BCG vaccine*					
	*No*	0 (0)	0 (0)	0 (0)	
	*Yes*	20 (100)	9 (100)	11 (100)	
*Smoker*					
	*No*	5 (25.0)	3 (33.3)	2 (18.2)	0.617
	*Yes*	15 (75.0)	6 (66.7)	9 (81.8)	
*Quit smoking*					
	*No*	5 (45.5)	2 (50.0)	3 (42.9)	0.999
	*Yes*	6 (54.5)	2 (50.0)	4 (57.1)	
*Passive smoker*					
	*No*	6 (46.2)	2 (33.3)	4 (57.1)	0.592
	*Yes*	7 (53.8)	4 (66.7)	3 (42.9)	
*Hypertension*					
	*No*	5 (26.3)	4 (44.4)	1 (10.0)	0.119
	*Yes*	14 (73.47)	5 (55.6)	9 (90.0)	
*Diabetes Mellitus*					
	*No*	15 (78.9)	7 (77.8)	8 (80.0)	0.667
	*Yes*	4 (21.1)	2 (22.2)	2 (20.0)	
*Benign prostatic hyperplasia (males)*					
	*No*	7 (77.8)	3 (60.0)	4 (100.0)	0.278
	*Yes*	2 (22.2)	2 (40.0)	0 (0)	
*Paint handling*					
	*No*	18 (94.7)	7 (87.5)	11 (100)	0.421
	*Yes*	1 (5.3)	1 (12.5)	0 (0)	
*Pesticide handling*					
	*No*	18 (90.0)	8 (88.9)	10 (90.9)	0.999
	*Yes*	2 (10.0)	1 (11.1)	1 (9.1)	
*Family history of neoplasia*					
	*No*	10 (52.6)	3 (33.3)	7 (70.0)	0.179
	*Yes*	9 (47.4)	6 (66.7)	3 (30.0)	
*Pathological Findings*					
	*Ta*	6 (30.0)	5 (55.6)	1 (9.1)	0.068
	*T1 low grade*	1 (5.0)	0 (0)	1 (9.1)	
	*T1 high grade*	12 (60.0)	4 (44.4)	8 (72.7)	
	*Carcinoma in situ*	1 (5.0)	0 (0)	1 (9.1)	
*Number of lesions*					
*Average* ± SD		2.60 ± 2.68	3.29 ± 2.99	1.00 ± 0.00	0.383
*Median (min–max)*		1 (1–8)	1 (1–8)	1 (1–1)	

Note: a. Totals may differ due to missing data.

There was no significant relationship between sex (*p* = 0.999). None of the patients had a history of previous tuberculosis. Approximately half of the patients have family members with a history of various neoplasms, with 66.7% in the controls and 30% in the vaccinated patients (*p* = 0.179).

There was no relationship between interventions and smoking. The control had 66.7% smokers, while the intervention had 81.8% (*p* = 0.617). This also applies to the variables quitting smoking, where 54.5% of patients claimed to have done so, and passive smoking, which is claimed by 53.8%. The median number of lesions was 1 for both.

Few patients handled paint (5.3%) and pesticides (10%), thus showing no relationship, with *p* values of 0.421 and 0.999, respectively. In the control, 55.6% of patients had hypertension, while 90% had it in the intervention. The test does not yield a significant *p*-value (*p* = 0.199). For the diabetes mellitus comorbidity, there’s a near marginal distribution between control and intervention, namely 21.1%.

When observed, benign prostate hyperplasia in men showed no relationship (*p* = 0.278) despite the apparent different percentage incidence between control and intervention, with 40% in the unvaccinated and 0% in the vaccinated patients.

Ta tumors were found in approximately 56% of patients in the control but only 9.1% in the vaccinated (*p* = 0.068), while 72.7% of vaccinated patients had T1 high-grade tumors compared with 44.4% in the unvaccinated patients.

There was no distribution difference between the IPSS and the intervention, as shown in Table 2. The control patients’ median value was zero at all time points, and the intervention had a median value of 2. At all time points, there were some outliers, but this is potentially due to a specific issue related to the patient’s underlying disease, such as benign prostatic hyperplasia.

**Table 2:** International prostatic symptoms score (IPSS) of included patients

	$Total^{a}$	Placebo	Intradermal BCG		
	n (%)	n (%)	n (%)	*p* ^+^	p*
*Total*	20 (100)	9 (45)	11 (55)		
*T0*					
*Average* ± SD	5.70 ± 9.79	6.22 ± 11.59	5.27 ± 8.62	0.710	0.217
*Median (min–max)*	1.5 (0–35)	0 (0–35)	2 (0–27)		
*T1*					
*Average* ± SD	5.70 ± 10.02	5.78 ± 11.69	5.63 ± 9.02	0.456	
*Median (min–max)*	0.5 (0–35)	0 (0–35)	2 (0–29)		
*T2*					
*Average* ± SD	6.35 ± 10.12	5.79 ± 11.69	6.81 ± 9.20	0.295	
*Median (min–max)*	1.5 (0–35)	0 (0–35)	2 (0–28)		
*T3*					
*Average* ± SD	7.50 ± 10.99	7.33 ± 11.76	7.63 ± 10.91	0.412	
*Median (min–max)*	2 (0–35)	0 (0–35)	2 (0–35)		
*T4*					
*Average* ± SD	6.35 ± 10.03	7.11 ± 11.63	5.72 ± 9.05	0.503	
*Median (min–max)*	2 (0–35)	0 (0–35)	2 (0–300)		

Note: a. Totals may differ due to data not shown. b. *Comparison by Friedman test between times points (dependent test). c. ^+^Comparison by Mann-Whitney test between placebo and intervention.

A test was also carried out to detect a potential difference in score distribution between time points, thus comparing patients with themselves. We found no significant differences, with a *p*-value of 0.217.

This pattern of patients not presenting symptoms was maintained in subsequent follow-ups. Notably, at the fourth follow-up visit (T3), the main observation was a scar at the vaccination site, which occurred in 50% of patients from the intervention.

**Figure 3:**
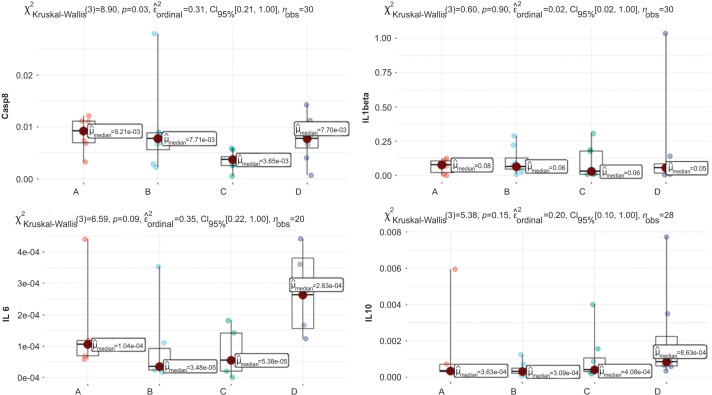
Graphs of Casp8, IL6, IL1beta, and IL10 quantification. Clinically relevant time points: Control, Intradermal BCG, Intravesical BCG, and Intradermal + Intravesical BCG are represented by A, B, C, and D, respectively.

**Figure 4:**
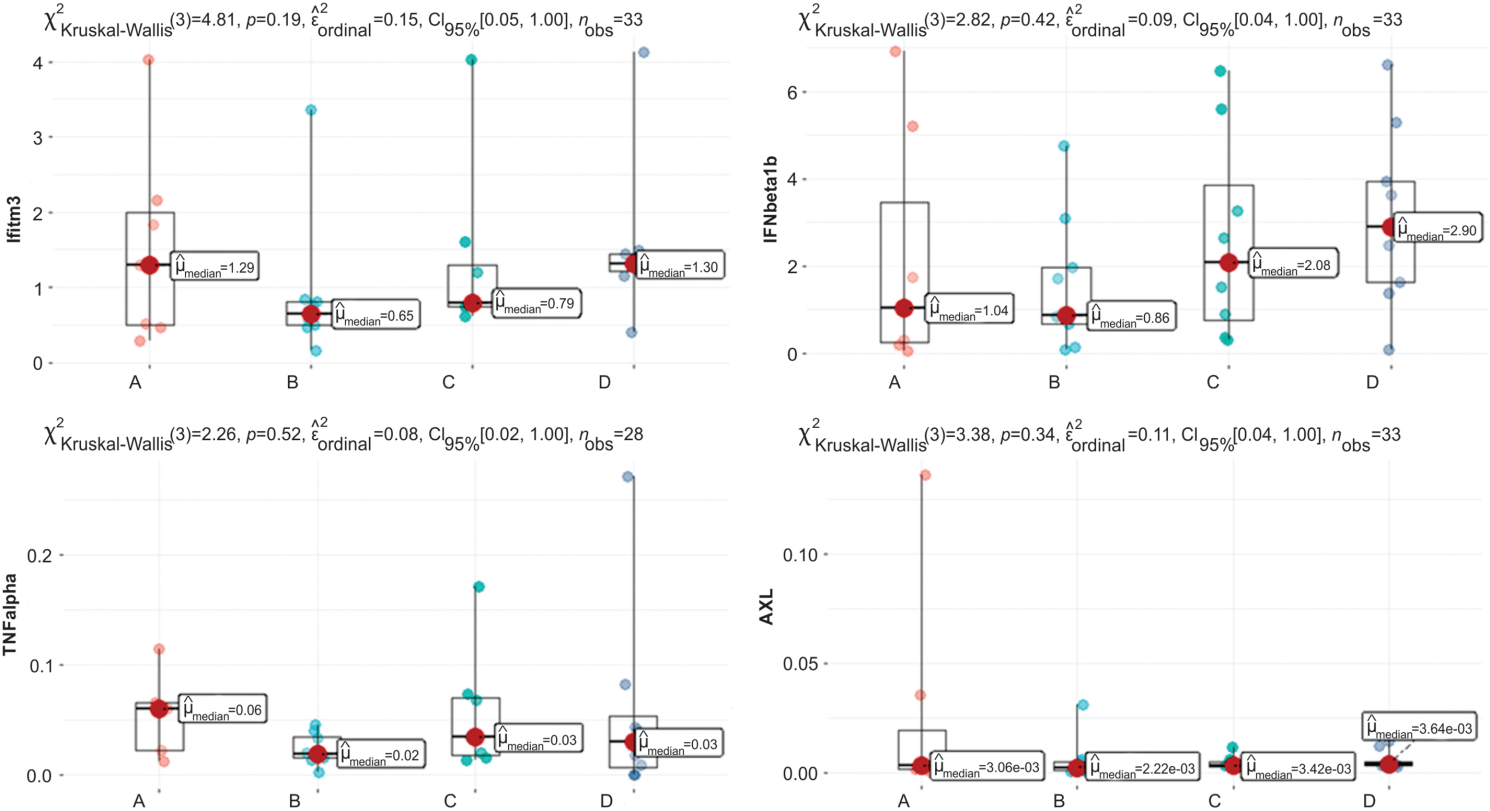
Graphs of Ifitm3, IFNβ1b, TNF-α, and AXL quantification. Clinically relevant time points: Control, Intradermal BCG, Intravesical BCG, and Intradermal + Intravesical BCG are represented by A, B, C, and D, respectively.

For blood analysis results, the clinically relevant time points Control (n = 9), Intradermal BCG (n = 11), Intravesical BCG (n = 9), and Intradermal + Intravesical BCG (n = 11) are represented by A, B, C, and D, respectively, in Figs. 3 and 4.

In Fig. 3, we see a difference in the distribution of the variable Casp8, *p* = 0.03, considering a significance level of 5%. When analyzing multiple comparison tests, we observed no significant results. Still, there was a trend between A and C (*p* = 0.054), where A has a median value of 0.00921, whereas C has a lower median value of 0.00365.

The distribution of variables IL1beta and IL2RA (Fig. 3) is not significantly different, with *p* = 0.60 and *p* = 0.035, respectively. We note that the median values between patients are close for both variables.

For variable IL6 (Fig. 3**)**, the graph shows that Kruskal-Wallis’s test reveals a trend, with *p* = 0.09. Still, in multiple comparisons, the *p* values found are *p* > 0.22 for all multiple comparisons, which leads us to conclude that there is no distribution difference between clinically relevant scenarios.

There was no difference in data distribution for the following variables: IL10, Ifitm3, TNF-α, IFNβ1b, and AXL (Fig. 4). This shows that neither vaccine intervention nor time points change gene and interleukin data distribution based on RNA.

## Discussion

This study has shown that it is safe to perform intradermal BCG priming before administering intravesical BCG. This approach does not increase the systemic and urinary symptoms related to the standard treatment in the Brazilian population.

The only clinical difference observed between the control and intervention patients was the presence of a local reaction, typical of the intradermal vaccine, which tends to trigger an inflammatory process. This aligns with our previous experience regarding intradermal BCG safety in convalescent COVID patients [27].

A change in serum IFITM3, IL1-BETA, IL2 RA, IL 6, IL 10, TNF-α, IFN-β, AXL, and CASPASE-8 was not demonstrable between the control and intervention during vesical therapy or within the same patient over time.

In 1976, Morales designed the first treatment protocol for bladder cancer with intravesical and intradermal BCG simultaneously [28]; later, the Southwest Oncology Group (SWOG) group validated the treatment with the exclusive use of intravesical BCG. However, in 1982, Brosman [29], in a 61-patient study, demonstrated the non-inferiority in terms of efficacy of the intravesical BCG as a single therapy. Over subsequent decades, several studies have shown an elevation in urinary cytokines, such as IL-2, IL-8, IL-10, and TNF-α, in oncology patients during therapy with the bacillus [30–32].

A study in rats showed higher and faster T-cell recruitment to the bladder with intravenous BCG priming, which, given the increased recurrence-free survival rate in PPD-positive patients [33], warrants a more thorough and robust investigation of systemic immune response preceding intravesical administration. Therefore, in 2019, Ji et al. administered intradermal BCG to 9 non-muscular invasive bladder cancer patients 21 days before the start of the induction phase and, through analysis of peripheral blood, found increased stimulation of Tγδ cells, which cooperate with NK cells in mediating BCG antitumoral toxicity [34].

The bacillus is known to be internalized in the bladder, particularly by tumor cells, which activates a complex innate and adaptive immune response. Furthermore, it produces a very particular type of response, namely a heterologous trained immune response, which is “learned” by the innate immune system and seems to play a central role in the antitumor and antiviral roles attributed to the vaccine.

This analysis suggests that the serum expression of these cytokines does not play a significant role in antitumoral immunomodulation in non-muscle invasive bladder cancer. However, further investigation into systemic priming is essential based on the studies mentioned, which highlight a potential link between systemic priming, increased stimulation of Tγδ cells, and lower cancer rates in patients vaccinated with BCG during childhood. Specifically, research should explore BCG administration earlier than three weeks before intradermal priming, focusing on analyzing other cytokines and the tumor microenvironment.

### Limitations

Despite this being a prospective, randomized, single-blind study, with analysis at several points in the induction therapy, certain limitations must be taken into account: The relatively small number of patients enrolled (the result of the COVID-19 pandemic and limited availability of BCG in Brazil during the study period), absence of a comparative assessment of cytokines in the tumoral microenvironment, as well as the absence of follow-up records of recurrence or disease progression in these patients.

Current results obtained in a tuberculosis endemic region, where BCG vaccination is mandatory, might not be extrapolated to populations of non-endemic contexts.

Understanding the processes involved in the body’s immune response to cancer provides foundational knowledge for future studies in bladder cancer therapy and supports optimizing the limited supply of BCG worldwide.

## Conclusion

This prospective, randomized, placebo-controlled study determined the safety of a systemic boost with BCG immunization. It showed that there was no increase in the side effects of administering Onco-BCG after systemic priming regarding symptoms and the IPSS questionnaire.

Also, no significant serum variation of IFITM3, IL1-BETA, IL2 RA, IL 6, IL 10, TNF-α, IFN-β, AXL, and CASPASE-8 was observed following administration of intravesical Onco-BCG over 6 weeks, even when these patients were subjected to systemic priming 15 days before the start of intravesical applications. Still, this finding cannot be extrapolated to other populations or to the tumor microenvironment and urine status; additional research is needed, including the long-term oncological response. The Brazilian tuberculosis-endemic status, where BCG vaccination is mandatory, might have affected the results.

## Data Availability

The collected nonpersonal patient data and material transfer for this study are available after the lead contact communicates and agrees.

## Appendix A

**Table A1:** Targeted cytokines rational (8–24 manuscript references)

Cytokine	Action
CASPASE 8	This is a protease caspase with a key regulatory role in programmed cell death and in the inflammatory process. When it cleaves Caspase 3, it induces cell apoptosis, and on GSDMD it causes pyroptosis, whereas when it cleaves RIPK1 and RIPK3, it inhibits necroptosis. This cytokine also regulates the inflammasomes’ response to certain viral and bacterial pathogens [9,10].
IFITM3	A transmembrane protein induced by interferon 3. In addition to its antiviral role, which is well-established in scientific literature, it also acts in restricting mycobacterial growth and plays a role in endosomal maturation mediated by IFITM in its antimycobacterial activity [11,12].
IL1-β	A paracrine cytokine is one of the main markers of a non-specific inflammatory immune response induction. It is produced by virtually all nucleated cell types, particularly monocytes, macrophages, and dendritic cells [13,14].
IL2 RA	Membrane receptor that binds the alpha subunit of IL-2. Regulates NK-cell and lymphocyte activation, as well as regulatory T cells [15,16].
IL6	It is related to innate and adaptive immune responses. By modulating T and B lymphocytes, it is the main activator of the immune response during the acute phase [17,18].
IL10	Produced by T and B lymphocytes, monocytic cells, natural killer cells, dendritic cells, eosinophils, and neutrophils, this interleukin is responsible for an anti-inflammatory response and immune suppression by suppressing the expression of Th1 cytokines, class II MHC, and costimulatory molecules in macrophages. It can impair NF-κB activity and is involved in the regulation of the JAK-STAT signaling pathway [19,20].
TNF-α	It may be found to be a transmembrane protein, as well as a soluble cytokine, and its receptors are TNFR1 and TNFR2. A pro-inflammatory cytokine that regulates the pathogenesis of tuberculosis and leads to tumor cell apoptosis TNF-α [21,22].
IFN-β	A polypeptide, typically produced by fibroblasts, with antiviral and antiproliferative effects. IFN-β reduces antigen presentation and T-cell proliferation, changes cytokine and matrix metalloproteinase (MMP) expression, in addition to having antiangiogenic and antiproliferative effects [23,24].
AXL	Tyrosine kinase receptor from the TAM subfamily. TOLL-like receptor inhibition, natural killer-cell activation, and thrombotic response modulation [25].

**Table A2:** Assays used in qPCR

Gene name	IDT DNA technologies assay	Ref. seq	Exon boundary
*Axl*	Hs.PT.56a.19269785	NM_001699(2)	15–17
*Il1β*	Hs.PT.58.1518186	NM_000576(1)	1–3
	**Thermo Fisher Assay**		
*Casp8*	Hs01594281_m1	NM_001137667.1	8–9
*Il10*	Hs00961622_m1	NM_000572.2	4–5
*Il2ra*	Hs00907777_m1	NM_000417.2	2–3
*Il6*	Hs00174131_m1	NM_000600.4	3–4
*Ifnβ*	Hs01077958_s1	NM_002176.3	1–1
*Ifitm3*	Hs03057129_s1	NM_021034.2	2–2
*Tnfα*	Hs00174128_m1	NM_000594.3	3–4
*Ppia*	Hs04194521_s1	NM_001300981.1	6–6

Note: Gene names, assay codes, reference sequences, and regions were used to construct the assays purchased from Thermo Fischer Scientific or IDT DNA Technologies. *Ppia* was used as a reference gene (endogenous control).
